# Pharmacokinetic Comparison of Tylvalosin Tartrate Nanocrystal Suspension and Soluble Powder in Broiler Chickens After Oral and Intravenous Administration

**DOI:** 10.3390/vetsci12101004

**Published:** 2025-10-17

**Authors:** Ao Lin, Yanzhe Qing, Yani Gu, Jingjie Huang, Xinxin Ma, Jiancheng Li

**Affiliations:** 1 Beijing Key Laboratory of Detection Technology for Animal-Derived Food Safety, Beijing Laboratory for Food Quality and Safety, College of Veterinary Medicine, China Agricultural University, Beijing 100193, China; la000531@163.com (A.L.); s20243051078@cau.edu.cn (Y.Q.); guyanihe@163.com (Y.G.); h15081883875@163.com (J.H.); maxinxin0925@163.com (X.M.); 2 Department of Veterinary Medicine, China Agricultural University, No. 2 Yuan Ming Yuan Western Road, Hai Dian District, Beijing 100193, China

**Keywords:** tylvalosin, broilers, pharmacokinetics, UPLC–MS/MS

## Abstract

This study was performed to investigate and compare the pharmacokinetic characteristics of tylvalosin tartrate in broiler chickens following oral administration of a nanocrystal suspension (PO-NM) or a soluble powder formulation (PO-SP), with intravenous administration (IV) of tylvalosin tartrate serving as the reference standard. A total of 30 healthy broiler chickens were randomly allocated into three groups (PO-NM, PO-SP, and IV; *n* = 10). Tylvalosin was administered at a dose of 25 mg/kg body weight (BW), and blood samples were collected at multiple time points from 0 to 24 h post-administration. Plasma concentrations of tylvalosin were quantified using a validated ultra-performance liquid chromatography–tandem mass spectrometry (UPLC-MS/MS) method, and pharmacokinetic parameters were calculated using non-compartmental analysis. The results showed no significant differences in the terminal elimination half-life (t_1/2λz_) and mean residence time (MRT) between the two oral formulations. However, the time to maximum concentration (Tmax) of PO-NM (0.71 ± 0.09 h) was significantly shorter than that of PO-SP (1.42 ± 0.18 h) (*p* < 0.05), while the maximum plasma concentration (Cmax) of PO-NM (255.52 ± 111.88 ng/mL) was markedly higher than that of PO-SP (120.45 ± 45.82 ng/mL) (*p* < 0.05). Furthermore, the absolute bioavailability (F) of PO-NM (15.73 ± 4.29%) showed a modest increase compared with PO-SP (11.45 ± 4.66%); however, this difference did not reach statistical significance. Collectively, these findings demonstrate that the PO-NM formulation achieved faster absorption, higher peak plasma levels, and greater systemic exposure compared with PO-SP, without significantly altering the elimination process. Overall, nanoparticle formulation appears to enhance the oral pharmacokinetic performance of tylvalosin in broiler chickens, potentially reducing residue risks and offering substantial application value in poultry medicine.

## Simple Summary

This study evaluated how tylvalosin behaves in chickens when given as two different oral formulations—a nanocrystalline suspension and a soluble powder—compared with intravenous administration. Thirty healthy broilers received a single dose of 25 mg/kg, and blood samples were analyzed by UPLC-MS/MS. The nanocrystalline suspension was absorbed faster and reached higher blood levels than the soluble powder. Although the overall bioavailability of tylvalosin remained low, the nanocrystalline formulation provided modest improvement, suggesting potential benefits in reducing dosage and drug residues in poultry.

## 1. Introduction

Avian mycoplasmosis is regarded as one of the most significant economic challenges in the global poultry industry, with *Mycoplasma gallisepticum* (MG) and *Mycoplasma synoviae* (MS) being the two most detrimental pathogens [1]. These organisms cause chronic respiratory disease and infectious synovitis in poultry. MG primarily targets the respiratory and reproductive systems, whereas MS is more often linked to synovitis. Co-infections are common and frequently result in chronic respiratory disease [2]. Both pathogens can be transmitted horizontally and vertically, and they are widely distributed in poultry populations worldwide [3]. Infections with MG and MS result in substantial reductions in production performance, such as decreased growth rate and poor feed conversion in broilers [2]; reduced egg production and inferior eggshell quality in laying hens [4]; and increased embryonic mortality and hatch failure in breeder flocks [2]. Collectively, these impacts contribute to considerable economic losses for the global poultry industry.

Antimicrobial therapy remains the most effective means of controlling MG- and MS-induced mycoplasmosis, with macrolides, tetracyclines, and fluoroquinolones showing strong activity in vitro and in vivo [5]. However, long-term antibiotic use has led to resistance in MG and MS, while recent studies show that tylvalosin, a novel macrolide, has much lower MICs than other agents [6]. This finding highlights the superior efficacy of tylvalosin among the tested antimicrobials.

For macrolides, pharmacokinetic/pharmacodynamic (PK/PD) integration is key to interpreting pharmacokinetic data. Their antimicrobial activity is primarily concentration-dependent with prolonged post-antibiotic effects [7]. In poultry, macrolides accumulate in respiratory tissues and macrophages, sustaining exposure at Mycoplasma infection sites even when plasma levels decline. Thus, pharmacokinetic parameters such as Cmax, AUC, and oral bioavailability (F) are directly linked to therapeutic outcomes and provide a rational basis for evaluating new formulations.

Tylvalosin is a novel, broad-spectrum, third-generation macrolide antibiotic derived from tylosin A through fermentation with a thermotolerant Streptomyces strain. It is structurally modified by acetylation at the 3-hydroxyl position and isovaleryl substitution at the 4′-hydroxyl position, mediated by acetyltransferase and isovaleryltransferase, respectively [8]. The antimicrobial mechanism of tylvalosin involves specific binding to the 50S ribosomal subunit of susceptible bacteria, thereby inhibiting the translocation of tRNA at the aminoacyl-receptor site and interrupting the elongation of nascent peptide chains, ultimately blocking protein synthesis [9]. Tylvalosin exhibits potent activity against *Mycoplasma* spp., *Spiroplasma* spp., most Gram-positive bacteria (including *Staphylococcus aureus*, *Streptococcus pyogenes*, *Streptococcus pneumoniae*, and *Corynebacterium pyogenes*), as well as certain Gram-negative bacteria. Clinically, it is widely used in the treatment of respiratory and enteric infections in swine and poultry [10].

In veterinary medicine, increasing attention has been directed toward the development and application of nanoscale drug formulations. Nanocarriers have emerged as efficient tools capable of enhancing and modifying the pharmacokinetic and pharmacodynamic properties of therapeutic agents [11]. Previous studies have demonstrated that nanotechnology-based formulations can improve drug stability, reduce toxicity, and facilitate targeted absorption, thereby enhancing overall therapeutic efficacy [12], However, research on the pharmacokinetics of tylvalosin in nanoscale formulations remains limited. Therefore, investigating the pharmacokinetic behavior of tylvalosin nanocrystal formulations is of particular importance for improving therapeutic outcomes and mitigating the risk of antimicrobial resistance.

Therefore, the present study was designed to compare the pharmacokinetic profiles of tylvalosin tartrate in broiler chickens following a single administration of 25 mg/kg body weight, delivered either orally as a nanocrystal suspension (PO-NM) or as a soluble powder (PO-SP), with intravenous administration serving as the reference. The findings are expected to provide valuable reference data for the future development of nanoscale veterinary drug formulations. In particular, this research may help to clarify how nanoparticle modification influences the absorption and systemic exposure of tylvalosin in poultry, offering insights into improving therapeutic efficacy, optimizing dosage regimens, and potentially reducing the risk of antimicrobial resistance and drug residues in food-producing animals.

## 2. Materials and Methods

### 2.1. Chemicals and Reagents

Tylvalosin standard (CAS 63409-12-1; purity ≥ 90%) was purchased from Macklin (Shanghai, China). Tylvalosin tartrate soluble powder was supplied by Qilu Animal Health Products Co., Ltd. (Jinan, Shandong, China), with each gram of powder containing 250 mg of tylvalosin as the active ingredient. Acetylisovaleryltylosin tartrate (CAS 63428-13-07) was obtained from Widely (Wuhan, Hubei, China). The tylvalosin tartrate nanocrystalline suspension was prepared and characterized by another research group within our laboratory. HPLC-grade acetonitrile (ACN) and formic acid (FA) were purchased from Thermo Fisher Scientific (Macquarie Park, North Ryde, NSW, Australia). Water was purified using a Milli-Q water purification system (Merck Millipore, Burlington, Middlesex County, MA, USA).

### 2.2. Experimental Animals

Thirty healthy broiler chickens (body weight 2.4–3.1 kg, equal sex ratio) were used in this study. Prior to the experiment, all birds underwent a one-week acclimatization period. The chickens were housed in cages (60 × 60 × 80 cm; four birds per cage) at the animal experimental facility of China Agricultural University. The housing environment was maintained at 22 ± 1 °C, with good ventilation and adequate lighting. All animals were fed a standard antibiotic-free diet and provided with water ad libitum. The experimental protocol was ethically approved by the Animal Breeding and Use Committee of China Agricultural University (AW01405202-2-03) (Beijing, China).

### 2.3. Experimental Design and Sample Collection

All broiler chickens were randomly and evenly assigned into three groups (IV, PO-NM, and PO-SP; n = 10 per group), with efforts made to maintain an approximately equal sex ratio within each group. To simulate practical drug use conditions, birds were provided with feed and water ad libitum before, during, and after dosing. Body weight was accurately recorded immediately prior to administration. The PO-NM group received a single oral gavage of tylvalosin tartrate nanocrystal suspension at a dose of 25 mg/kg body weight. The PO-SP group received a single oral gavage of tylvalosin tartrate soluble powder at the same dosage, while the IV group was administered an equivalent single intravenous dose via the wing vein. Blood samples (~1.0 mL) were collected from the wing vein into 2 mL anticoagulant tubes at the following time points: pre-dose (0 h), 0.083 h (IV only), 0.167 h, 0.25 h, 0.5 h, 0.75 h, 1 h, 1.5 h, 2 h, 4 h, 8 h, 12 h, and 24 h post-dose. Samples were immediately transferred into 1.5 mL centrifuge tubes and centrifuged at 4000 rpm for 10 min. The plasma supernatant was carefully separated and stored at −20 °C until analysis.

### 2.4. Sample Preparation

Stored plasma samples were thawed at room temperature. 200 μL of each plasma sample were mixed with 600 μL of acetonitrile in a centrifuge tube and vortexed for 5 min. The mixture was then centrifuged at 8000 rpm for 15 min at 4 °C. The supernatant was filtered through a 0.22 μm microporous membrane and transferred to an autosampler vial added into an autosampling product for UPLC–MS/MS detection.

### 2.5. UPLC-MS/MS Conditions

Plasma samples, after pretreatment, were analyzed using a UPLC–MS/MS system. Chromatographic separation was performed on an ACQUITY UPLC BEH Shield RP18 column (1.7 μm, 2.1 × 50 mm; Waters, Milford, MA, USA) maintained at 30 °C, with the autosampler temperature set at 17 °C. The mobile phase consisted of 0.1% formic acid in water (A) and 0.1% formic acid in acetonitrile (B), delivered at a flow rate of 0.3 mL/min. The gradient elution program was as follows: 0–0.6 min, 10% B; 0.6–1.5 min, 10–80% B; 1.5–3.0 min, 80% B; 3.0–3.1 min, 80–10% B; and 3.1–4.0 min, 10% B. Mass spectrometric detection was carried out on a positive electrospray ionization (ESI^+^) source operating in multiple reaction monitoring (MRM) mode. The precursor ion was *m*/*z* 1042.6, and the product ions were *m*/*z* 109.1 and 174.1. Quantification was based on the transition *m*/*z* 1042.6 → 109.1, with a cone voltage of 35 V and a collision energy of 40 eV.

### 2.6. Standards Preparation and Method Validation

A stock solution of tylvalosin (1 mg/mL) was prepared in acetonitrile and stored at −20 °C until use. Working solutions (0.05–10 μg/mL in acetonitrile) were prepared by serial dilution of the stock solution. Calibration standards were obtained by spiking 20 μL of the working solution into 180 μL of blank chicken plasma, resulting in final plasma concentrations within the calibration range. Accuracy and precision were evaluated using quality control (QC) samples at four concentrations (5, 10, 500, and 800 ng/mL). Eight replicates at each concentration were analyzed over three consecutive days to assess intra- and inter-day variability. The limit of detection (LOD) and limit of quantification (LOQ) were determined based on signal-to-noise (S/N) ratios, with LOD defined as S/N ≥ 3 and LOQ defined as S/N ≥ 10.

### 2.7. Data Analysis

Following quantification of tylvalosin concentrations in plasma samples, pharmacokinetic analysis was performed using a non-compartmental model with WinNonlin software (version 8.3.4; Certara, Pharsight, Mountain View, CA, USA). Results are presented as mean ± standard deviation (SD).

## 3. Results

### 3.1. Validation of Analytical Methods

The extraction method used in this study demonstrated high reproducibility and showed good linearity over the concentration range of 5–1000 ng/mL for tylvalosin, with correlation coefficients (r^2^) consistently greater than 0.99. The recovery of tylvalosin from spiked plasma samples ranged from 88.75% to 101.61%. Intra-day and inter-day coefficients of variation (CVs) were 1.31–8.25% and 1.47–13.23%, respectively. The limit of detection (LOD) and limit of quantification (LOQ) for tylvalosin were determined to be 2 ng/mL and 5 ng/mL, respectively. All validation results met the acceptance criteria recommended by the US FDA and EMA bioanalytical method validation guidelines, which require accuracy within ±15% (±20% at the LLOQ) and precision with CV ≤ 15% (≤20% at the LLOQ). These findings confirm that the developed method is reliable and suitable for the quantitative determination of tylvalosin in chicken plasma.

### 3.2. Pharmacokinetics

**Figure 1:**
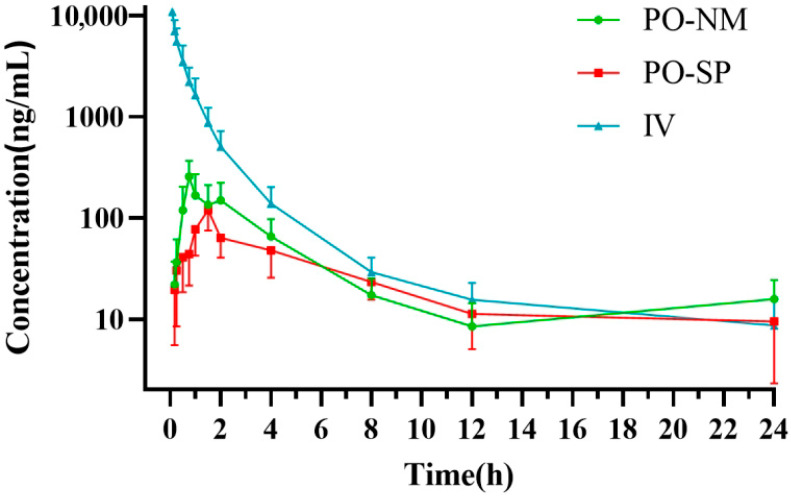
Drug concentration-time curves in plasma after administration. Note: Data is presented as Mean ± SD.

All 30 broiler chickens remained in stable condition throughout the experiment, and no abnormal clinical signs such as diarrhea, vomiting, or lethargy were observed during the 24 h study period following drug administration. Figure 1 illustrates the plasma concentration–time profiles of tylvalosin in the three groups (PO-NM, PO-SP, and IV), while Table 1 summarizes the corresponding pharmacokinetic parameters. Tylvalosin was detectable in plasma in all three groups up to 24 h post-administration.

Pharmacokinetic results demonstrated significant differences between the two oral formulations. First, Tmax was significantly shorter in the PO-NM group compared to the PO-SP group (0.71 ± 0.09 h vs. 1.42 ± 0.18 h) (*p* < 0.05), indicating that PO-NM enters systemic circulation more rapidly. Additionally, Cmax was significantly higher in the PO-NM group than in the PO-SP group (255.52 ± 111.88 ng/mL vs. 120.45 ± 45.82 ng/mL) (*p* < 0.05), indicating higher absorption rates. AUClast and AUC0–∞ values in the PO-NM group (918.90 ± 354.99 h·ng/mL and 1040.31 ± 377.33 h·ng/mL, respectively) were numerically higher than those in the PO-SP group (731.95 ± 374.28 h·ng/mL and 839.85 ± 426.58 h·ng/mL, respectively). Although this increase suggests a trend toward greater systemic exposure and oral absorption with the nanoformulation, the differences did not reach statistical significance. However, the elimination-related parameters λz, t_1/2λz_, and MRT showed no significant differences between the two groups, indicating that the two formulations exhibit similar clearance and distribution pharmacokinetic characteristics in vivo. The absolute bioavailability (F) of PO-NM (15.73 ± 4.29%) was numerically higher than that of PO-SP (11.45 ± 4.66%). However, consistent with the non-significant differences observed in AUClast and AUC0–∞ between the two oral formulations, this increase in F did not reach statistical significance.

**Table 1:** Pharmacokinetic parameters after administration.

Parameter	Units	PO-NM	PO-SP	IV
λz	1/h	0.11 ± 0.03	0.09 ± 0.03	0.16 ± 0.09
t_1/2λz_	h	6.54 ± 2.07	6.68 ± 1.49	4.26 ± 1.34
Tmax	h	0.71 ± 0.09	1.42 ± 0.18	-
Cmax	ng/ml	255.52 ± 111.88	120.45 ± 45.82	-
AUClast	h·ng/ml	918.90 ± 354.99	731.95 ± 374.28	6737.17 ± 2375.36
AUC0–∞	h·µg/mL	1040.31 ± 377.33	839.85 ± 426.58	6833.51 ± 2454.11
AUMC	h^2^·µg/mL	10.48 ± 6.27	10.33 ± 9.43	13.86 ± 12.25
MRT	h	5.80 ± 1.92	6.64 ± 1.43	1.44 ± 0.23
F	%	15.73 ± 4.29	11.45 ± 4.66	-
CL	L/h/Kg	-	-	4.44 ± 1.77
Vz	L/Kg	-	-	35.99 ± 23.56
Vss	L/kg	-	-	5.55 ± 1.58

Abbreviations: λ_z_, the first-order rate constant associated with the terminal phase; t_1/2λz_, terminal half-life; AUC0-∞, the area under the drug concentration–time curve from the time of administration to infinity; AUMC, the area under first-order moment curve from administration to infinity; MRT, mean residence time; V_Z_, volume of distribution; Vss, steady-state volume of distribution; CL, total body clearance; Tmax, the time to reach peak concentration; Cmax, peak concentration; F, absolute bioavailability. AUClast, area under concentration/time curve from 0 to last point.

## 4. Discussion

This study compared the pharmacokinetic characteristics of tylvalosin nanocrystal suspension and soluble powder in broiler chickens, using intravenous administration as the reference. The results indicated that the nanocrystal formulation had a significant effect on the absorption process of the drug, while its influence on the elimination process was limited.

The peak concentration of 120.45 ± 45.82 ng/mL was reached at 1.42 ± 0.18 h after oral administration of the tylvalosin tartrate soluble powder. At the same oral dose (25 mg/kg BW), this Cmax was within the range of previous studies in broilers (143.96 ± 11.11 ng/mL [13] to 287.12 ± 253.07 ng/mL [14]). Such variability may arise from differences in breeds, age, body weight, feeding conditions, and experimental designs [15]. Nevertheless, these values remain within the same order of magnitude. Meanwhile, the Tmax after oral administration of the soluble powder to broilers was 1.42 ± 0.18 h, consistent with which was similar to previous results (Tmax of 1.88 ± 0.69 h [14], 1.20 ± 0.37 h [16], 2.31 ± 0.12 h [13], and 2.09 ± 0.14 h [17]). Few pharmacokinetic studies on tylvalosin have been reported in species other than broilers; one study in turkeys found a Tmax of 2.00 ± 0.00 h [18]. Moreover, when examining other macrolide antibiotics such as tylosin, previous studies revealed species-dependent differences in Tmax: 6.00 ± 0.00 h in lactating goats [19], 2.00 ± 0.00 h in ducks [20], 1.33 ± 0.18 h in broilers [21], 1.50 h in 12-week-old turkeys [22], and 3.00 ± 0.44 h in Beagles [23]. These data suggest that avian species have similar Tmax values after oral administration of tylvalosin. Of interest, broilers showed significantly shorter Tmax (0.71 ± 0.09 h) and higher Cmax (255.52 ± 111.88 ng/mL) after oral administration of the tylvalosin tartrate nanocrystalline suspension compared to the oral soluble powder. This improvement is likely attributable to the faster dissolution of the drug from the smaller particles, resulting in more rapid absorption. In addition, nanocrystals can prolong gastrointestinal residence time and adhere to the intestinal mucosa, thereby enhancing epithelial penetration and further increasing the absorption rate [24]. For example, nanocrystals of apigenin were prepared by a supercritical anti-solvent process, and after a single oral administration of apigenin to rats, the nanocrystals showed significantly lower Tmax and a Cmax 3.6 times higher than that of the those of crude apigenin particles in rats after a single oral dose [25].

In contrast, no significant differences were observed between the two groups with respect to elimination-related parameters, including t_1/2λz_, λz, and MRT. These findings indicate that once tylvalosin has entered the systemic circulation, the two formulations behave in essentially the same way in terms of distribution and elimination. This outcome is expected, because the intrinsic clearance and distribution of a drug are determined by its physicochemical properties and the host’s metabolic and excretory systems, which are not altered by the formulation [26]. In this study, we found little difference in t_1/2λz_ between the PO-NM and PO-SP dosing groups, 6.54 ± 2.07 and 6.68 ± 1.49 h, respectively, both higher than that of the IV dosing group, 4.26 ± 1.34 h. Observed differences in t_1/2λz_ between the two oral formulations and the IV group are most plausibly methodological rather than reflecting true changes in elimination. Formulation primarily alters gastrointestinal absorption (dissolution, permeability), not intrinsic clearance or distribution. When the absorption rate constant approaches or is lower than the elimination rate constant, the terminal slope is governed by absorption (flip-flop kinetics), yielding an apparently longer or shorter t_1/2λz_ despite unchanged clearance [27]. In contrast to previous broiler studies, it was much lower than that of the oral dosing at the same dose (25 mg/kg BW), 11.7 h [13], higher than 1.86 ± 0.83 h for the same dose administered orally but similar to its t_1/2λz_ 5.06 ± 3.13 h for the lower dose group (5 mg/kg BW) [14]. Also, it was much higher than the t_1/2λz_ of turkeys administered orally at the same dose (0.95 ± 0.15) [18]. This suggests that the mode of administration, dose, animal species and individual differences in tylvalosin affect its elimination half-life. Furthermore, unlike the pharmacokinetic study of mangiferin’s nanoformulation in rabbits (1.5-fold prolongation of t_1/2λz_) [28], nanocrystalline suspensions of tylvalosin tartrate did not prolong the elimination half-life, which is similar to the results of florfenicol’s nanoformulation in broiler chickens [29], which suggests that particle size, morphology, and selection of stabilizers for the nanoformulation may affect the drug’s s t_1/2λz_ [30].

The PO-SP group had an F of 11.45 ± 4.66%, which was similar to that of broilers given a 10 mg/kg oral dose of tylvalosin (13.74%) [21], higher than that reported for broilers given 25 mg/kg (3.04%) [14], and much lower than that of turkeys given 25 mg/kg (53.3%) [18]. The PO-NM group had a higher F (15.73 ± 4.29%) than the PO-SP group. This slight increase in F may be attributed to the faster and more complete dissolution of the nanocrystalline formulation in the gastrointestinal tract, which enables greater absorption within the limited intestinal residence time and reduces drug loss due to incomplete dissolution [31]. By accelerating dissolution, nanocrystals facilitate more efficient absorption and thereby contribute to the observed improvement in absolute bioavailability. Numerous studies have shown that nanocrystalline formulations can effectively improve the bioavailability of orally administered drugs, such as the oral administration of florfenicol nanocrystalline formulations to broiler chickens, which increased F by approximately 2-fold [29], Ginkgolide B by about 5-fold [32], and the anticancer drug Venetoclaxtigaol by about 2-fold [33]. However, in the present study, the improvement in F was not significant, probably because the soluble powder formulation also dissolves and absorbs the vast majority of the drug over a longer period of time [25], and the total amount absorbed by the two preparations was similar.

## 5. Conclusions

This study is the first to compare the pharmacokinetics of tylvalosin nanocrystal suspension and soluble powder following single oral administration in broiler chickens. The results demonstrated that, compared with the soluble powder, the nanocrystal suspension significantly shortened Tmax, increased Cmax, and moderately improved bioavailability. The enhanced absorption of tylvalosin nanocrystals suggests the potential to reduce dosing frequency or lower the required dose, thereby minimizing drug usage and decreasing potential residues in animal-derived food products. Future studies could be further strengthened in the following aspects: (1) inter-individual variability in broiler pharmacokinetics was relatively high; subsequent studies should include larger sample sizes and investigate the influence of age, sex, and body weight; (2) optimization of the nanocrystal formulation to further enhance bioavailability, enabling dose reduction without compromising efficacy, which may also help mitigate the risk of antimicrobial resistance.

## Data Availability

The original contributions presented in this study are included in the article. Further inquiries can be directed to the corresponding author.
